# Time-Dependent Response of a Recycled C&D Material-Geotextile Interface under Direct Shear Mode

**DOI:** 10.3390/ma14113070

**Published:** 2021-06-04

**Authors:** Fernanda Bessa Ferreira, Paulo M. Pereira, Castorina Silva Vieira, Maria de Lurdes Lopes

**Affiliations:** CONSTRUCT, Faculty of Engineering, University of Porto, Rua Dr. Roberto Frias, 4200-465 Porto, Portugal; fbf@fe.up.pt (F.B.F.); paulmppereira@gmail.com (P.M.P.); lcosta@fe.up.pt (M.d.L.L.)

**Keywords:** recycled construction and demolition materials, geosynthetics, high-strength geotextile, direct shear tests, long-term interface response, stress relaxation, geosynthetic-reinforced structures

## Abstract

Geosynthetic-reinforced soil structures have been used extensively in recent decades due to their significant advantages over more conventional earth retaining structures, including the cost-effectiveness, reduced construction time, and possibility of using locally-available lower quality soils and/or waste materials, such as recycled construction and demolition (C&D) wastes. The time-dependent shear behaviour at the interfaces between the geosynthetic and the backfill is an important factor affecting the overall long-term performance of such structures, and thereby should be properly understood. In this study, an innovative multistage direct shear test procedure is introduced to characterise the time-dependent response of the interface between a high-strength geotextile and a recycled C&D material. After a prescribed shear displacement is reached, the shear box is kept stationary for a specific period of time, after which the test proceeds again, at a constant displacement rate, until the peak and large-displacement shear strengths are mobilised. The shear stress-shear displacement curves from the proposed multistage tests exhibited a progressive decrease in shear stress with time (stress relaxation) during the period in which the shear box was restrained from any movement, which was more pronounced under lower normal stress values. Regardless of the prior interface shear displacement and duration of the stress relaxation stage, the peak and residual shear strength parameters of the C&D material-geotextile interface remained similar to those obtained from the conventional (benchmark) tests carried out under constant displacement rate.

## 1. Introduction

Over the last few decades, the application of geosynthetics as soil reinforcement elements in permanent earth structures, such as retaining walls, embankments, steep slopes, and bridge abutments has become a well-established technology. Among the advantages of geosynthetic-reinforced soil structures are the cost-effectiveness, relatively short construction time, possibility of using lower quality and/or locally available backfill materials, including recycled waste materials, flexibility, and ductility, as well as the adequate performance, even when constructed in seismic areas. For the internal stability analysis and safe design of such structures, the interaction properties at soil-geosynthetic interfaces are of the utmost importance [1,2,3]. Various experimental methods, such as the direct shear test, inclined plane test, pullout test, and in-soil tensile test have been developed to characterise soil-geosynthetic interaction, of which the pullout and direct shear tests are the most typically used [3,4,5,6,7,8,9,10].While the pullout test is a valuable method to examine the interaction between the soil and the geosynthetic, in the anchorage zone of geosynthetic-reinforced soil walls, slopes, and embankments, the direct shear test is better suited to analyse the soil-geosynthetic interface behaviour in cases where sliding of the soil mass on the reinforcement surface is likely to occur [2,3,7,11,12].

In recent years, the use of recycled construction and demolition (C&D) wastes in geotechnical engineering works (e.g., road and railway infrastructure and geosynthetic-reinforced structures) has been pointed out as a cost-effective and sustainable way of mitigating the environmental impacts arising from the overexploitation of natural resources, while also decreasing the waste disposal volumes in landfills [13,14,15,16,17,18,19]. In fact, vast amounts of C&D wastes are produced each year by the construction industry, which has raised significant environmental concerns at a global level. In the European Union, the construction industry is responsible for over 35% of total waste generation [20]. The vast amounts of C&D wastes generated, associated with their high valorisation potential, have led the European Commission to recognize these materials as a priority waste stream for reuse and recycling [21]. Therefore, the use of recycled C&D wastes, as an alternative backfill in geosynthetic-reinforced structures, would significantly contribute towards sustainability and environmental protection. However, a thorough understanding of the long-term response of such structures is required before they can be safely put into practice.

Geosynthetics, being polymeric materials, exhibit a time-dependent stress-strain behaviour (i.e., creep and stress relaxation) when subjected to tensile loads. The intrinsic viscoelastic nature of geosynthetics may affect the performance of the reinforced structures throughout their design lifetimes, and thereby, the long-term mechanical properties of geosynthetics and soil-geosynthetic interfaces are of great importance when these materials are used in permanent reinforcement applications. Several factors, such as the polymer type, loading rate, temperature, and soil confinement are susceptible to influence the time-dependent response of geosynthetics [22,23,24].

The long-term tensile behaviour of geosynthetics has been typically assessed by creep and creep rupture tests and, albeit less often, using stress relaxation tests [25,26,27,28,29,30,31,32,33]. This can be partly explained by the fact that current design guidelines are based upon creep test results, and thus do not require stress relaxation data, as well as the relatively higher complexity of stress relaxation testing [25]. While in creep testing, the applied tensile load is kept constant, and the resulting strains are measured over time, in a stress relaxation test the strain is kept constant through the reduction in the tensile load with time, which inevitably demands a sophisticated test device with feedback control.

Leshchinsky et al. [25] investigated the creep and stress relaxation behaviour of several typical geogrids used in reinforced soil walls and slopes. The geogrid specimens were subjected to initial loads corresponding to 40–80% of the ultimate short-term tensile strength, and the tests were conducted for a period of one month, or until rupture occurred (whichever was shortest). The authors found that the maximum potential stress relaxation was about 30% and 50% of the initial loads for polyester and high-density polyethylene geogrids, respectively.

Allen and Bathurst [34] analysed the reinforcement creep data, measured in 10 full-scale geosynthetic walls, and compared them with the creep rates obtained by unconfined (i.e., in-isolation) laboratory creep tests. They concluded that, in the majority of cases, the laboratory creep rates were identical, or greater than, the measured reinforcement creep rates in the full-scale walls, suggesting that in-isolation laboratory creep data generally produce conservative estimates of creep in walls. The authors also reported that, at the end of wall construction, the reinforcement appeared to be primarily exhibiting creep, with negligible stress relaxation. However, in the long-term, there was a trend towards reinforcement stress relaxation.

Recently, Costa and Zornberg [22] presented a novel laboratory test device suitable to evaluate the time-dependent deformation of geosynthetics experienced under soil confinement. Apart from soil-geosynthetic interaction tests, the apparatus enables in-isolation geosynthetic stress relaxation tests and soil-only tests to be performed under a constant strain rate. The soil-geosynthetic interaction tests, carried out on a sand and a polypropylene woven geotextile, were found to lead to increasing geotextile deformations, and decreasing reinforcement tension, over time. For the geotextile tested, the authors observed a reduction in time-dependent deformations under confinement conditions in comparison to those obtained by in-isolation creep tests. Their study also showed that the lower deformations, over time, obtained under confinement conditions occurred as a result of the load reduction throughout the test, rather than due to the effect of soil confinement.

The above literature review has shown that, although significant research has been conducted in the past to investigate the long-term response of geosynthetics, very limited studies have focused on the time-dependent interaction behaviour at soil-geosynthetic interfaces. In particular, to the best of the authors’ knowledge, no previous studies have been conducted to evaluate the time-dependent response of soil-geosynthetic interfaces under direct shear mode.

In view of the above, an innovative direct shear test method is used in this study to investigate the time-dependent phenomena (stress relaxation) taking place at the interface between a high-strength geotextile and a fine-grained recycled C&D material. A large-scale direct shear testing programme, involving conventional and multistage tests, is carried out under varying normal stresses, shear displacements at the onset of the stress relaxation stage, and stress relaxation periods. A comparison is then made between the interface shear stress-shear displacement response, and the vertical deformation of the specimens, obtained from both test methods, to examine the effect of time on the interface shear strength properties.

## 2. Materials and Methods

### 2.1. Materials

**Figure 1:**
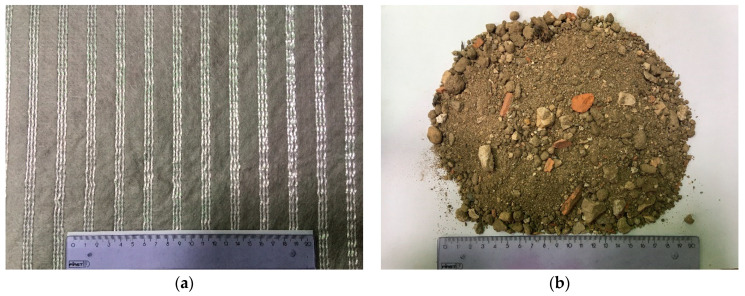
Materials used in the study: (**a**) High-strength geotextile; (**b**) Recycled C&D material.

A commercially-available uniaxial high-strength geotextile, also termed as a geocomposite reinforcement, was used in this study (Figure 1a). This geotextile consists of high-modulus polyethylene terephthalate yarns attached to a continuous filament nonwoven polypropylene geotextile, with a nominal tensile strength of 40 kN/m and corresponding elongation of 10% (machine direction), according to the manufacturer specifications. Due to its in-plane permeability, this geotextile reduces excess pore-water pressure and is therefore suitable for backfill materials with high fines content.

The recycled C&D material (Figure 1b) is a fine-grained material resulting from the recycling process carried out at a Portuguese specialized recycling plant, which involves several steps, including the removal of metals and light contaminants (e.g., plastics, rubbers, cork, foams, etc.,) in a trommel and the separation by granulometric fractions through conveyor belts and multiple sieves. The batch of the recycled material used in the study was produced from C&D wastes coming mainly from maintenance and rehabilitation works of residential buildings, demolition of masonry fences and recovering of C&D wastes from illegal dumps.

**Figure 2:**
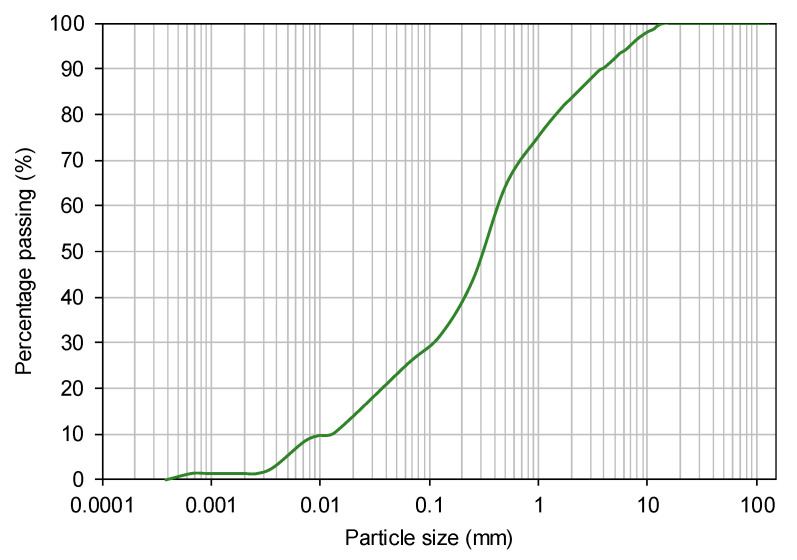
Particle size distribution curve of the recycled C&D material.

The relative proportion of constituents of recycled C&D aggregates may be estimated on the basis of the European Standard EN 933-11:2011 [35] (Table 1). However, this standard is applicable to coarse recycled aggregates, whereby the particles whose diameter is lower than 4 mm are not considered. Taking into account that a large proportion of the recycled C&D material under investigation consists of particles passing the 4 mm sieve (see Figure 2), but the manual sorting of these particles is humanly impossible, an alternative classification was also included in Table 1. Assuming that the unsorted particles can be classified as soil, it can be considered that this recycled material is mostly composed of soil with some particles of concrete, mortars, unbound and hydraulically bound aggregates.

The particle size distribution of the recycled C&D material was evaluated by sieving and sedimentation in accordance with the EN 933-1:2012 [36] and CEN ISO/TS 17892-4:2016 [37] Standards (Figure 2). The optimum compaction parameters (i.e., optimum moisture content, w_opt_ and maximum dry unit weight, γ_dmax_) were determined using the standard Proctor test, following the EN 13286-2:2010 [38]. The main physical properties of this recycled material are presented in Table 2.

The evaluation of the leaching behaviour of recycled C&D materials to be used in geosynthetic-reinforced structures is of great relevance to investigate the potential for groundwater contamination and the presence of chemical substances that could induce the degradation of geosynthetics. In this study, laboratory leaching tests were carried out on the recycled C&D material following the European Standard EN 12457-4:2002 [39] to determine the concentration of each contaminant. The obtained values were then compared with the acceptance criteria for inert landfill according to the European Council Decision 2003/33/EC [40] (Table 3).

It can be concluded that, except for sulphate whose concentration (1200 mg/kg of dry matter) exceeded the maximum value established by the European legislation for inert landfill (1000 mg/kg of dry matter), all the pollutants were significantly below the respective limits. The relatively high concentration of sulphate in mixed recycled aggregates is usually attributed to the presence of gypsum drywall [41]. It is noteworthy that according to the European legislation [40], if the waste material exceeds the threshold value for sulphate, it can still be considered as meeting the acceptance criteria as long as the leaching does not exceed 6000 mg/kg at a liquid to solid (L/S) ratio of 10 L/kg.

### 2.2. Large-Scale Direct Shear Apparatus and Test Procedures

The direct shear tests on the recycled C&D material-geotextile interface were conducted using a large-scale direct shear test apparatus that is suitable to characterise the direct shear behaviour of soils, soil-geosynthetic, and geosynthetic-geosynthetic interfaces under monotonic and cyclic loading conditions. The equipment comprises a shear box (including upper and lower boxes), a support structure, five hydraulic actuators and respective fluid power unit, an electric cabinet and a number of internal and external transducers. The upper and lower boxes are 600 mm long × 300 mm wide × 150 mm deep and 800 mm long × 340 mm wide × 100 mm deep, respectively. The apparatus is capable of performing direct shear tests with constant or reduced contact area during shearing, depending on whether a rigid base or, alternatively, a rigid ring is installed in the lower box, respectively. Further details about the large-scale direct shear test apparatus can be found in previous publications [12,42].

**Figure 3:**
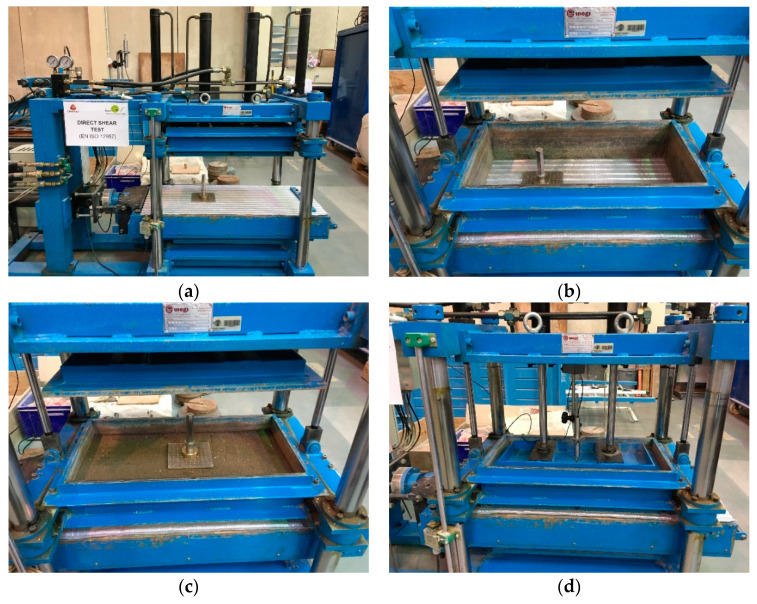
Large-scale direct shear apparatus and test procedures: (**a**) Geotextile specimen fixed to the lower box; (**b**) Upper box positioned over the geotextile; (**c**) Upper box filled with compacted recycled C&D material; (**d**) Direct shear box ready for testing.

As per the European Standard EN ISO 12957-1: 2018 [43], direct shear tests to characterise soil-geosynthetic interfaces shall be performed by fixing the geosynthetic to a rigid, horizontal support in the lower shear box, except for tests on interfaces involving geogrids with large apertures (>15 mm) and a high percentage of openings (>50% of the overall surface of the specimen), in which a soil support (i.e., lower box filled with soil) can alternatively be employed. Following the recommendations of the aforementioned standard, a rigid base was installed inside the lower box, thereby enabling constant contact area direct shear tests. The geotextile specimen was fixed to the lower box using a rigid bar with bolts to avoid any relative displacement between the specimen and the support (Figure 3a). The upper box was positioned allowing a gap of about 1 mm between its base and the geotextile top surface (Figure 3b). As shown in Figure 3c, four 25 mm thick layers of C&D material were then compacted inside the upper box to a dry unit weight of 17.1 kN/m^3^ (corresponding to 90% of the maximum dry unit weight) at the optimum moisture content, as determined by the standard Proctor test [38].

The direct shear tests were conducted under varying normal stress levels (*σ_n_*) ranging from 25 kPa to 150 kPa. The required normal stress was applied to the test specimen by a rigid plate with pressure-controlled double acting linear actuators and the vertical displacements were recorded using an external displacement transducer (i.e., a Linear Variable Differential Transformer, LVDT) positioned at the rigid plate centre (Figure 3d). The values of the applied normal stress, vertical displacement of the rigid plate, horizontal displacement of the lower box, and shear stress mobilised at the C&D material-geotextile interface were continuously monitored during the tests.

### 2.3. Test Programme

The test conditions investigated in this study are summarized in Table 4. The test programme involved 20 large-scale direct shear tests on a recycled C&D material-geotextile interface (5 conventional and 15 multistage direct shear tests). The conventional (i.e., benchmark) direct shear tests were performed at a constant displacement rate of 1 mm/min and up to a maximum shear displacement of 60 mm. In order to examine the time-dependent stress-strain behaviour of the C&D material-geotextile interface, an innovative multistage direct shear test procedure was implemented including three different stages: first, a constant displacement rate of 1 mm/min was applied until a prescribed horizontal (shear) displacement (*d_r_*) was reached; in the second stage, the displacement of the lower box was restrained for a specific period of time (*t_r_*), while the stress decay associated with stress relaxation was continuously measured; in the final stage, the test proceeded again at a constant displacement rate (1 mm/min) until an horizontal displacement of 60 mm was reached.

The effect of the magnitude of shear displacement, at which the stress relaxation phase started, was investigated by defining two different displacement levels (i.e., *d_r_* = 1/3 *d_max_* and *d_r_* = 2/3 *d_max_*, where *d_max_* is the shear displacement corresponding to the maximum shear stress in the benchmark test conducted under the same normal stress). The period of time during which the shear box was kept restrained varied between 30 and 120 min. All of the direct shear tests were conducted under normal stresses (*σ_n_*) ranging from 25 to 150 kPa. To assess the repeatability of results, the tests under *σ_n_* = 100 kPa were repeated.

## 3. Results and Discussion

### 3.1. Direct Shear Behaviour under Constant Displacement Rate

**Figure 4:**
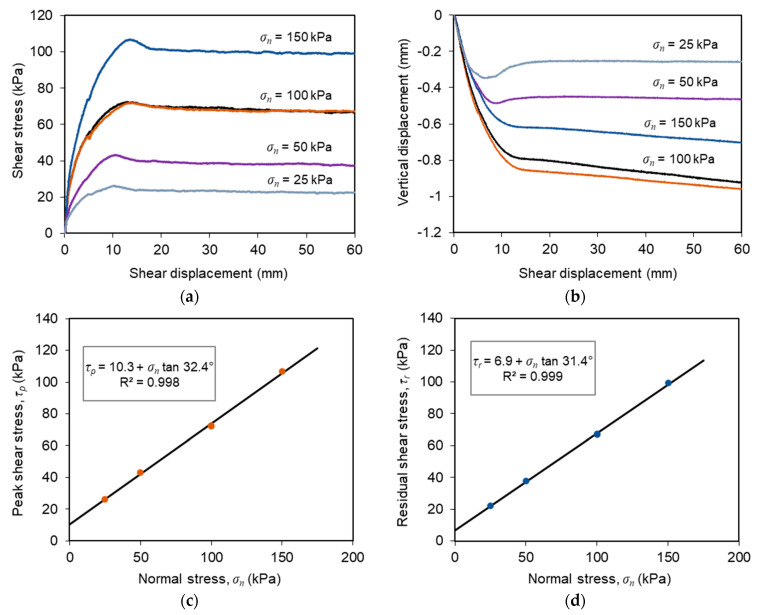
Results from conventional large-scale direct shear tests (tests T-1 to T-5): (**a**) Shear stress-shear displacement curves; (**b**) Vertical displacement of the loading plate centre; (**c**) Peak shear strength envelope; (**d**) Residual shear strength envelope.

Figure 4 shows the results from tests T-1 to T-5 carried out to evaluate the direct shear behaviour of the C&D material-geotextile interface subjected to a constant displacement rate of 1 mm/min under normal stresses ranging from 25 to 150 kPa. The evolutions of shear stress as a function of shear displacement, presented in Figure 4a, show well-defined peak shear strengths irrespective of the applied normal stress. Beyond the peak value, the shear strength decreased with any additional shear displacement until a steady state was reached (residual shear strength). As expected, both the peak and residual shear strengths increased with the normal stress. Furthermore, the peak strength was attained at shear displacements that increased progressively with the normal stress.

From the vertical displacements, measured by the LVDT installed at the loading plate centre (Figure 4b), it can be concluded that the specimens essentially exhibited a contractile behaviour during shearing (i.e., settlement). The vertical deformation was more pronounced at the early stage of shear displacement and tended to increase with the normal stress. For the lower normal stresses (i.e., *σ_n_* = 25 and 50 kPa), the vertical deformation tended to stabilise once the interface strain softening response was completed. In contrast, when higher normal stresses were applied (*σ_n_* = 100 and 150 kPa), the vertical settlement increased continuously with shear displacement until the end of the test.

The peak and residual shear strengths mobilised at the studied interface when subjected to a constant displacement rate, and different normal stresses are illustrated in Figure 4c,d, respectively, along with the corresponding linear best fits (Mohr-Coulomb strength envelopes) and respective equations. The interface peak shear strength can be characterised by a friction angle (*δ*) of 32.4° and adhesion (c_a_) of 10.3 kPa, whereas the residual shear strength can be characterised by a slightly lower friction angle of 31.4° and adhesion of 6.9 kPa.

### 3.2. Time-Dependent Interface Behaviour

**Figure 5:**
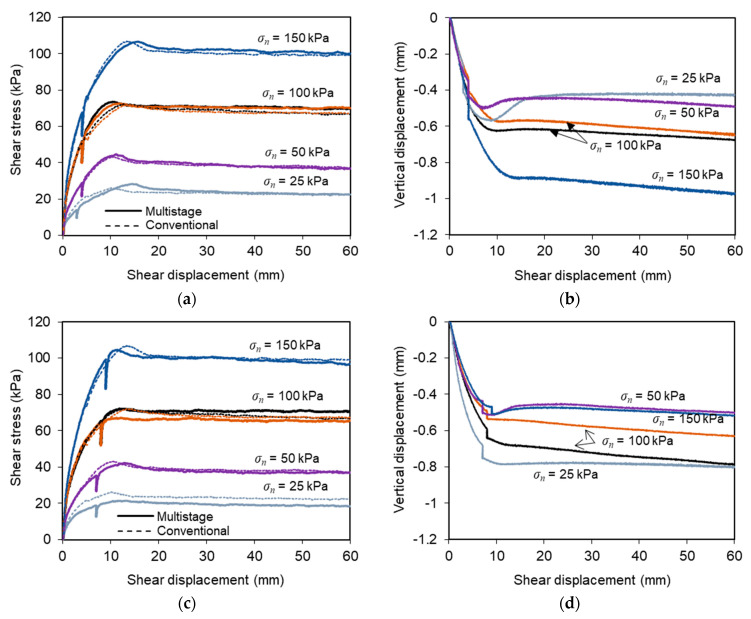

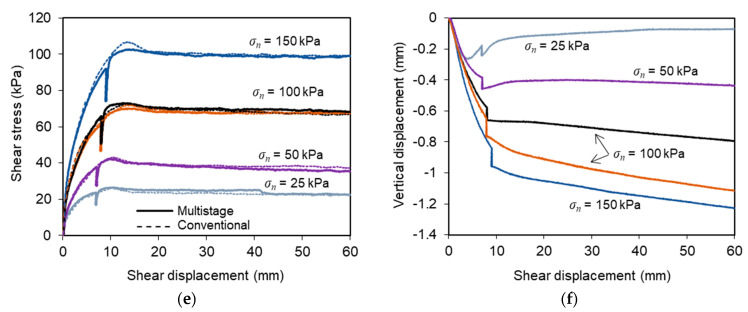
Time-dependent interface response under varying test conditions: (**a**,**b**) *d_r_/d_max_* = 1/3 and *t_r_* = 30 min (tests T-6 to T-10); (**c**,**d**) *d_r_/d_max_* = 2/3 and *t_r_* = 30 min (tests T-11 to T-15); (**e**,**f**) *d_r_/d_max_* = 2/3 and *t_r_* = 120 min (tests T-16 to T-20).

Figure 5 presents the shear stress-shear displacement curves, as well as the evolution of the vertical displacements obtained in multistage tests T-6 to T-20. Figure 5a,b show the results from tests in which the shear box was restrained for a period (*t_r_*) of 30 min at the shear displacement *d_r_* = 1/3 *dmax*. The results obtained when a larger shear displacement *d_r_* was considered are shown in Figure 5c–f, where Figure 5c,d are associated with *t_r_* = 30 min and Figure 5e,f are associated with *t_r_* = 120 min. The shear stress versus shear displacement curves, obtained in the benchmark direct shear tests, are also included in Figure 5a,c,e for comparison purposes.

The evolution of shear stresses throughout the multistage tests followed similar trends to those observed in the corresponding benchmark tests, except for the stress reduction recorded during the period in which the shear displacement was restrained (Figure 5a,c,e). This stress reduction can be attributed to stress relaxation. Once the shear displacement was restarted, the shear stress-shear displacement curves exhibited high stiffness, and hence the shear stress values quickly attained those obtained prior to the stress relaxation stage.

The vertical displacements recorded during the multistage tests (Figure 5b,d,f) showed that the specimens experienced some additional vertical settlement during the stress relaxation stage, which was mobilised at a relatively slow rate. The specimens mainly exhibited a contractive behaviour during shearing, with the maximum settlement at the end of the tests ranging from 0.07 to 1.23 mm under different test conditions. These values are relatively similar to those reached at the end of the conventional tests performed under constant displacement rate and varying normal stresses (0.27 to 0.97 mm).

**Figure 6:**
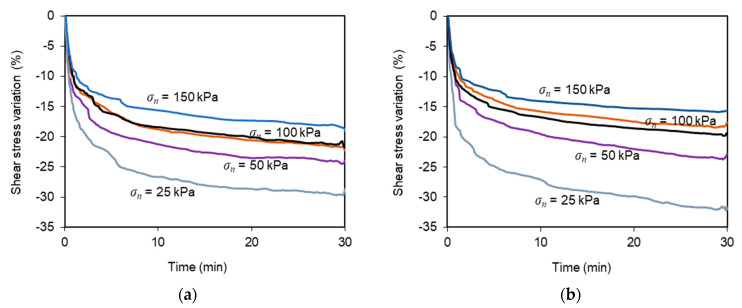

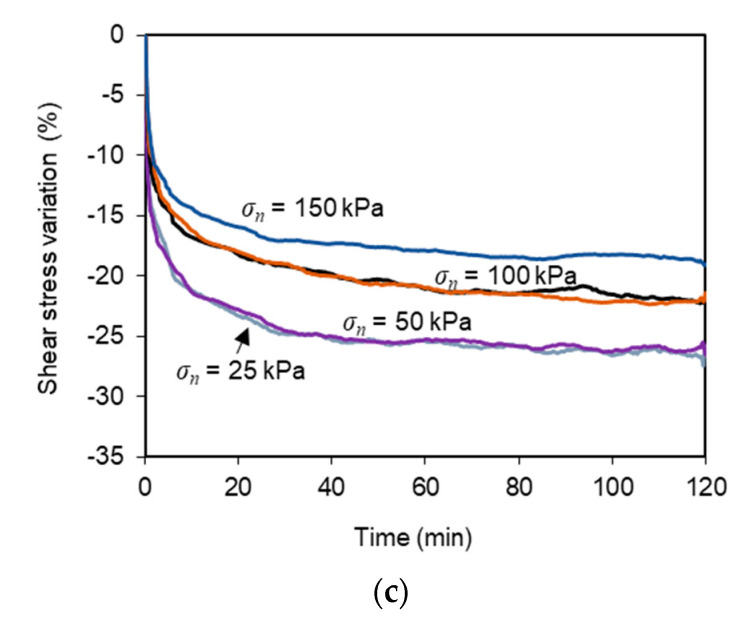
Percent variation of shear stresses over time during the stress relaxation stage: (**a**) *d_r_/d_max_* = 1/3 and *t_r_* = 30 min (tests T-6 to T-10); (**b**) *d_r_/d_max_* = 2/3 and *t_r_* = 30 min (tests T-11 to T-15); (**c**) *d_r_/d_max_* = 2/3 and *t_r_* = 120 min (tests T-16 to T-20).

Figure 6 plots the percent variation of shear stress recorded during the multistage tests when the shear box was restrained from any movement for 30 min (tests T-6 to T-15; Figure 6a,b) or 120 min (tests T-16 to T-20; Figure 6c). Figure 6a is related to a displacement *d_r_* = 1/3 *d_max_*, whereas Figure 6b,c are related to a displacement *d_r_* = 2/3 *d_max_*.

Regardless of the shear displacement *d_r_*, the shear stress decreased at a faster rate at the beginning of the stress relaxation stage, and then, the rate of stress decay reduced progressively over time. Beyond the initial 30 min, the additional stress reduction was almost negligible (Figure 6c). It can also be observed that the percent reduction of shear stress, recorded during the stress relaxation stage, became more pronounced as the normal stress was progressively decreased. For instance, for *d_r_/d_max_* = 1/3 and *t_r_* = 30 min (Figure 6a), the shear stress decreased by up to 30% under the normal stress of 25 kPa, whereas an 18% stress reduction was observed under the normal stress of 150 kPa. This finding suggests that the time-dependent phenomena, taking place at the studied interface, are more pronounced under lower normal stress values.

The displacement *d_r_* did not significantly affect the stress relaxation observed in these tests. In fact, for *d_r_* = 2/3 *d_max_* and *t_r_* = 30 min (Figure 6b), the percent reductions of the shear stress were similar to those attained under the lower *d_r_* value (e.g., 32% for *σ_n_* = 25 kPa and 16% for *σ_n_* = 150 kPa). When the stress relaxation period (*t_r_*) was increased to 120 min (Figure 6c), the stress reductions were generally slightly higher than those reached after *t_r_* = 30 min (Figure 6b).

### 3.3. Comparison of Interface Shear Strength Envelopes

**Figure 7:**
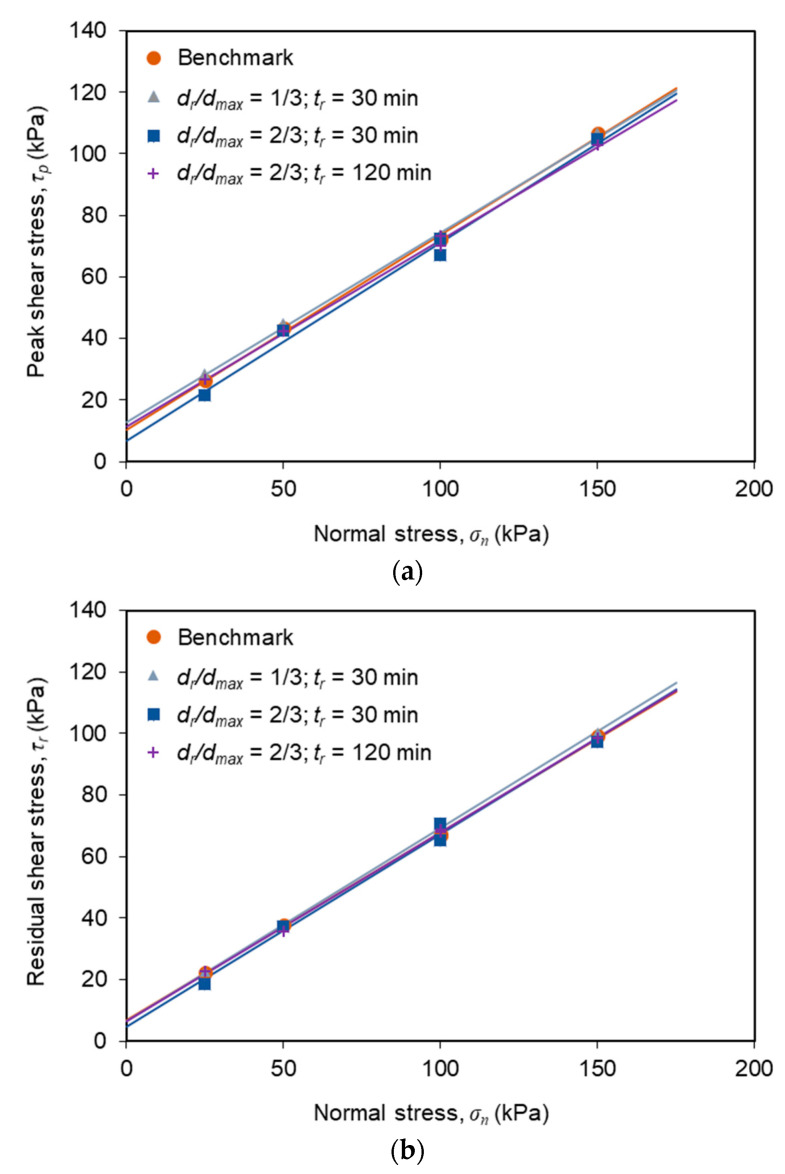
Interface shear strength envelopes from conventional (benchmark) and multistage tests: (**a**) Peak strength envelopes; (**b**) Residual strength envelopes.

The peak and residual shear strength envelopes (Mohr-Coulomb strength envelopes) of the C&D material-geotextile interface, which correspond to the best fit straight lines drawn through the maximum and residual shear stresses, obtained under different normal stress values, are plotted in Figure 7a,b, respectively. The interface shear strength parameters (i.e., adhesion, *c_a_* and friction angle, *δ*) for the different test conditions were then derived (Table 5).

Comparing the shear strength envelopes, determined from the benchmark and multistage tests, it becomes apparent that, for the conditions investigated in this study, the influence of stress relaxation on the interface peak and residual shear strength can be considered almost negligible. The values of the peak friction angle, obtained from the multistage tests, ranged from 31.2° to 32.8°, whereas the peak friction angle, obtained from the conventional tests, was equal to 32.4°. Regarding the corresponding interface adhesion, the values obtained from the multistage tests varied between 6.9 and 12.9 kPa, whereas the adhesion estimated from the benchmark test was 10.3 kPa. In turn, the residual interface friction angle and adhesion ranged from 31.6° to 32.2° and from 4.6 to 6.6 kPa in the multistage tests, respectively. Similar values were determined from the benchmark tests (31.4° and 6.9 kPa, respectively).

These results suggest that neither the stress relaxation period, nor the shear displacement at which stress relaxation occurs, are susceptible to affect the interface peak and residual shear strength, implying that the conventional direct shear tests can be considered suitable to characterise the long-term interface strength properties under direct shear mode.

It is noteworthy that the results obtained herein, for the C&D material-geotextile interface, are in good agreement with those reported in previous studies involving natural soils and a similar high-strength geotextile [12,44], as well as recycled C&D materials and other geosynthetics [14,45,46]. Previous studies carried out with similar recycled C&D materials have shown that, when properly compacted, these recycled materials can exhibit shear strength similar to, or even greater than, that of natural soils commonly used in the construction of geosynthetic-reinforced structures [13,45,47].

The aforementioned evidence supports the feasibility of using these fine-grained recycled C&D materials as a substitute backfill material in the construction of geosynthetic-reinforced structures, which would represent a valuable contribution towards the implementation of circular economy in the construction sector.

## 4. Conclusions

A series of large-scale direct shear tests was carried out in this study to characterise the time-dependent response of the interface between a recycled C&D material and a high-strength geotextile under direct shear mode. An innovative multistage direct shear test procedure was used, whereby the interface was subjected to stress relaxation after an initial period of monotonic loading at a constant displacement rate. The interface peak and residual shear strength parameters were compared with those from conventional direct shear tests to evaluate the effect of time on the interface shear strength properties. The following conclusions can be derived from the experimental results obtained in this study.

- The evolution of shear stress with shear displacement during the first and third phases of the multistage tests followed similar trends to those observed in the corresponding benchmark tests. During the period in which the shear box was restrained from any movement (*t_r_* = 30 or 120 min), the shear stress decreased progressively with time, which can be attributed to stress relaxation.
- In both the conventional and multistage direct shear tests, the specimens exhibited mainly a contractive behaviour during shearing. Throughout the stress relaxation stage, some additional vertical settlement was mobilised, albeit at a relatively slow rate. The cumulative vertical deformation at the end of the tests ranged from 0.27 to 0.97 mm in the conventional tests, and from 0.07 to 1.23 mm in the multistage tests.
- Regardless of the test conditions (i.e., normal stress, *σ_n_*, horizontal displacement, *d_r_* and duration, *t_r_* of the stress relaxation stage), the shear stress decreased at a faster rate at the beginning of the stress relaxation stage, after which the decreasing rate reduced progressively with time. Beyond the initial 30 min, the stress reduction became marginal.
- The shear stress decay, observed in the multistage tests, tended to increase with decreasing normal stress. The highest (32%) and lowest (16%) percent reductions of shear stress, at the end of the stress relaxation stage, were attained in tests conducted under the normal stresses of 25 and 150 kPa, respectively. The shear displacement at which the stress relaxation stage occurred (*d_r_*) did not significantly affect the respective percent reduction in shear stress.
- After the period of immobilization of the shear box, and once the shear displacement was restarted, the shear stress-shear displacement curves exhibited high stiffness, with the shear stress values rapidly reaching those mobilised immediately before the start of the stress relaxation stage.
- Based on the results from the conventional tests, the interface peak shear strength can be characterised by a friction angle of 32.4° and adhesion of 10.3 kPa, whereas the residual shear strength can be characterised by a friction angle of 31.4° and adhesion of 6.9 kPa. For the test conditions examined in this study, the effect of stress relaxation on the interface peak and residual shear strength was almost negligible, implying that the conventional large-scale direct shear tests can be considered suitable to evaluate the long-term interface strength properties under direct shear mode.

The results, presented herein, constitute part of a broader research project currently under development. It is worth mentioning that the conclusions presented refer to the geosynthetic and the recycled C&D material used in the study.

## Figures and Tables

**Table 1:** Constituents of the recycled C&D material.

Constituents	According to [35] *	Global Value
Concrete, concrete products, mortar, concrete masonry units, R_c_ (%)	16.0	1.6
Unbound aggregate, natural stone, hydraulically bound aggregate, R_u_ (%)	45.7	4.6
Clay masonry units, calcium silicate masonry units, aerated non-floating concrete, R_b_ (%)	3.7	0.4
Bituminous materials, R_a_ (%)	2.2	0.2
Glass, R_g_ (%)	0.3	0.0
Other materials, X ** (%)	32.1	3.2
Unsorted particles (%)	-	90
Floating particles, FL (cm^3^/kg)	3.8	-

* Only for particles above 4 mm; ** Materials that do not fall into the above categories (e.g., gypsum drywall, cork, non-floating wood and soils resulting from the washing process).

**Table 2:** Physical properties of the recycled C&D material.

Property	Value
D_10_ (mm)	0.01
D_50_ (mm)	0.33
D_60_ (mm)	0.45
Fines content (%)	26.5
C_U_	39.3
C_C_	2.4
γ_dmax_ (kN/m^3^) ^a^	19.0
w_opt_ (%) ^a^	11.3

^a^ Estimated from the standard Proctor test (EN 13286-2:2010 [38]).

**Table 3:** Results of laboratory leaching tests of the recycled C&D material and corresponding acceptance criteria.

Parameter	Value (mg/kg)	Acceptance Criteria—Inert landfill (mg/kg)
Arsenic, As	0.03	0.5
Lead, Pb	<0.3	0.5
Cadmium, Cd	<0.04	0.04
Chromium, Cr	<0.5	0.5
Copper, Cu	<0.25	2
Nickel, Ni	<0.3	0.4
Mercury, Hg	<0.01	0.01
Zinc, Zn	0.52	4
Barium, Ba	0.16	20
Molybdenum, Mo	0.03	0.5
Antimony, Sb	<0.01	0.06
Selenium, Se	0.02	0.1
Chloride, Cl	56	800
Fluoride, F	<2	10
Sulphate, SO_4_	1200	1000
Phenol index	<0.05	1
Dissolved Organic Carbon, DOC	<200	500
pH	8.6	-

**Table 4:** Direct shear test programme.

Test Number	Test Method	Normal Stress, *σ_n_* (kPa)	Displacement, *d_r_/d_max_*	Time, *t_r_* (min)
T-1	Conventional	25	N/A	N/A
T-2	Conventional	50	N/A	N/A
T-3	Conventional	100	N/A	N/A
T-4	Conventional	100	N/A	N/A
T-5	Conventional	150	N/A	N/A
T-6	Multistage	25	1/3	30
T-7	Multistage	50	1/3	30
T-8	Multistage	100	1/3	30
T-9	Multistage	100	1/3	30
T-10	Multistage	150	1/3	30
T-11	Multistage	25	2/3	30
T-12	Multistage	50	2/3	30
T-13	Multistage	100	2/3	30
T-14	Multistage	100	2/3	30
T-15	Multistage	150	2/3	30
T-16	Multistage	25	2/3	120
T-17	Multistage	50	2/3	120
T-18	Multistage	100	2/3	120
T-19	Multistage	100	2/3	120
T-20	Multistage	150	2/3	120

**Table 5:** Peak and residual interface shear strength parameters.

Test Method	*d_r_*/*d_max_*	*t_r_* (min)	Peak		Residual	
			*δ* (°)	*c_a_* (kPa)	*δ* (°)	*c_a_* (kPa)
Conventional	N/A	N/A	32.4	10.3	31.4	6.9
Multistage	1/3	30	31.7	12.9	32.2	6.6
Multistage	2/3	30	32.8	6.9	32.1	4.6
Multistage	2/3	120	31.2	11.5	31.6	6.5

## Data Availability

The data presented in this study are available on request from the corresponding author. The data are not publicly available due to privacy reasons.

## References

[1] Palmeira E.M. (2009). Soil–geosynthetic interaction: Modelling and analysis. Geotext. Geomembr..

[2] Lopes M.L. (2012). Soil-geosynthetic interaction. Handbook of Geosynthetic Engineering.

[3] Moraci N., Cardile G., Gioffrè D., Mandaglio M.C., Calvarano L.S., Carbone L. (2014). Soil Geosynthetic Interaction: Design Parameters from Experimental and Theoretical Analysis. Transp. Infrastruct. Geotechnol..

[4] Mendes M.J.A., Palmeira E.M., Matheus E. (2007). Some factors affecting the in-soil load–strain behaviour of virgin and damaged nonwoven geotextiles. Geosynth. Int..

[5] Liu C.-N., Ho Y.-H., Huang J.-W. (2009). Large scale direct shear tests of soil/PET-yarn geogrid interfaces. Geotext. Geomembr..

[6] Ezzein F.M., Bathurst R.J. (2014). A new approach to evaluate soil-geosynthetic interaction using a novel pullout test apparatus and transparent granular soil. Geotext. Geomembr..

[7] Ferreira F., Vieira C., Lopes M., Carlos D. (2015). Experimental investigation on the pullout behaviour of geosynthetics embedded in a granite residual soil. Eur. J. Environ. Civ. Eng..

[8] Ferreira F.B., Vieira C.S., Lopes M.L. Soil-geosynthetic interface strength properties from inclined plane and direct shear tests—A comparative analysis. Proceedings of the 6th Asian Regional Conference on Geosynthetics.

[9] Shaykhi P., Briançon L., Lajevardi S.H. (2020). Experimental evaluation of geosynthetics interface friction with a new procedure by using inclined plane. Innov. Infrastruct. Solut..

[10] Ferreira F.B., Vieira C.S., Lopes M.D.L. (2020). Pullout Behavior of Different Geosynthetics—Influence of Soil Density and Moisture Content. Front. Built Environ..

[11] Chen J.-F., Gu Z.-A., Rajesh S., Yu S.-B. (2021). Pullout Behavior of Triaxial Geogrid Embedded in a Transparent Soil. Int. J. Géoméch..

[12] Ferreira F.B., Vieira C.S., Lopes M.L. (2015). Direct shear behaviour of residual soil–geosynthetic interfaces–influence of soil moisture content, soil density and geosynthetic type. Geosynth. Int..

[13] Santos E.C., Palmeira E.M., Bathurst R.J. (2013). Behaviour of a geogrid reinforced wall built with recycled construction and demolition waste backfill on a collapsible foundation. Geotext. Geomembr..

[14] Vieira C.S., Pereira P.M., Lopes M.D.L. (2016). Recycled Construction and Demolition Wastes as filling material for geosynthetic reinforced structures. Interface properties. J. Clean. Prod..

[15] Vieira C.S., Ferreira F.B., Pereira P.M., Lopes M.D.L. (2020). Pullout behaviour of geosynthetics in a recycled construction and demolition material–Effects of cyclic loading. Transp. Geotech..

[16] Maghool F., Senanayake M., Arulrajah A., Horpibulsuk S. (2021). Permanent deformation and rutting resistance of demolition waste triple blends in unbound pavement applications. Materials.

[17] Pourkhorshidi S., Sangiorgi C., Torreggiani D., Tassinari P. (2021). Using Recycled Aggregates from Construction and Demolition Waste in Unbound Layers of Pavements. Sustainability.

[18] Vieira C.S., Pereira P., Ferreira F., Lopes M.D.L. (2020). Pullout Behaviour of Geogrids Embedded in a Recycled Construction and Demolition Material. Effects of Specimen Size and Displacement Rate. Sustainability.

[19] Jiménez J.R., Ayuso J., Agrela F., López M., Galvín A.P. (2012). Utilisation of unbound recycled aggregates from selected CDW in unpaved rural roads. Resour. Conserv. Recycl..

[20] European Commission (2020). EU Circular Economy Action Plan. https://ec.europa.eu/environment/circular-economy/.

[21] European Commission (2018). EU Construction & Demolition Waste Management Protocol. https://ec.europa.eu/growth/content/eu-construction-and-demolition-waste-protocol-0_en.

[22] Costa C.M.L., Zornberg J.G. (2021). Novel experimental techniques to assess the time-dependent deformations of geosynthetics under soil confinement. J. Rock Mech. Geotech. Eng..

[23] Sawicki A., Świdziński W. (1999). Unconfined Versus Confined Testing of Geosynthetics. Geosynth. Int..

[24] Becker L.D.B., Nunes A.L.L.D.S. (2015). Influence of soil confinement on the creep behavior of geotextiles. Geotext. Geomembr..

[25] Leshchinsky D., Dechasakulsom M., Kaliakin V.N., Ling H.I. (1997). Creep and stress relaxation of geogrids. Geosynth. Int..

[26] Bathurst R.J., Huang B.Q., Allen T.M. (1997). Interpretation of laboratory creep testing for reliability-based analysis and load and resistance factor design (LRFD) calibration. Geosynth. Int..

[27] Li A., Rowe R. (2001). Influence of Creep and Stress-Relaxation of Geosynthetic Reinforcement on Embankment Behaviour. Geosynth. Int..

[28] Kongkitkul W., Tatsuoka F., Hirakawa D. (2007). Creep rupture curve for simultaneous creep deformation and degradation of geosynthetic reinforcement. Geosynth. Int..

[29] Miyata Y., Bathurst R.J., Allen T.M. (2014). Reliability analysis of geogrid creep data in Japan. Soils Found..

[30] Kongkitkul W., Chantachot T., Tatsuoka F. (2014). Simulation of geosynthetic load–strain–time behaviour by the non-linear three-component model. Geosynth. Int..

[31] Pinho-Lopes M., Paula A.M., Lopes M.L. (2018). Long-term response and design of two geosynthetics: Effect of field installation damage. Geosynth. Int..

[32] Nuntapanich N., Kongkitkul W., Tatsuoka F., Jongpradist P. (2018). Prediction of creep behaviour from load relaxation behaviour of polymer geogrids. Geosynth. Int..

[33] Dias Filho J.L.E.D., Maia P., Xavier G.D.C. (2019). A short-term model for extrapolating unconfined creep deformation data for woven geotextiles. Geotext. Geomembr..

[34] Allen T., Bathurst R. (2002). Observed Long-Term Performance of Geosynthetic Walls and Implications for Design. Geosynth. Int..

[35] CEN (2009). Tests for Geometrical Properties of Aggregates—Part 11: Classification Test for the Constituents of Coarse Recycled Aggregate.

[36] CEN (2012). Tests for Geometrical Properties of Aggregates—Part 1: Determination of Particle Size Distribution–Sieving Method.

[37] CEN (2016). Geotechnical Investigation and Testing–Laboratory Testing of Soil—Part 4: Determination of Particle Size Distribution.

[38] CEN (2010). Unbound and Hydraulically Bound Mixtures—Part 2: Test Methods for Laboratory Reference Density and Water Content–Proctor Compaction.

[39] CEN (2002). Characterisation of Waste–Leaching–Compliance Test for Leaching of Granular Waste Material and Sludges—Part 4.

[40] European Commission, Council Decision 2003/33/EC (2003). Establishing Criteria and Procedures for the Acceptance of Waste at Landfills Pursuant to Article 16 of and Annex II to Directive.

[41] Jang Y.-C., Townsend T. (2001). Sulfate leaching from recovered construction and demolition debris fines. Adv. Environ. Res..

[42] Vieira C.S., Lopes M.L., Caldeira L. (2013). Sand-geotextile interface characterisation through monotonic and cyclic direct shear tests. Geosynth. Int..

[43] CEN (2018). Geosynthetics–Determination of Friction Characteristics—Part 1: Direct Shear Test.

[44] Ferreira F.B., Vieira C.S., Lopes M.L. Analysis of soil-geosynthetic interfaces shear strength through direct shear tests. Proceedings of the International Symposium on Design and Practice of Geosynthetic-Reinforced Soil Structures.

[45] Vieira C.S., Pereira P.M. (2017). Use of Mixed Construction and Demolition Recycled Materials in Geosynthetic Reinforced Embankments. Indian Geotech. J..

[46] Arulrajah A., Rahman M.A., Piratheepan J., Bo M.W., Imteaz M.A. (2014). Evaluation of Interface Shear Strength Properties of Geogrid-Reinforced Construction and Demolition Materials Using a Modified Large-Scale Direct Shear Testing Apparatus. J. Mater. Civ. Eng..

[47] Vieira C.S., Pereira P.M. (2015). Interface shear properties of geosynthetics and construction and demolition waste from large-scale direct shear tests. Geosynth. Int..

